# MicroRNA-155 enhances T cell trafficking and antiviral effector function in a model of coronavirus-induced neurologic disease

**DOI:** 10.1186/s12974-016-0699-z

**Published:** 2016-09-07

**Authors:** Laura L. Dickey, Colleen L. Worne, Jessica L. Glover, Thomas E. Lane, Ryan M. O’Connell

**Affiliations:** Department of Pathology, University of Utah School of Medicine, Salt Lake City, UT 84112 USA

**Keywords:** miR-155, Virus, Neuroinflammation, T cells, Demyelination

## Abstract

**Background:**

MicroRNAs (miRNAs) are noncoding RNAs that modulate cellular gene expression, primarily at the post-transcriptional level. We sought to examine the functional role of miR-155 in a model of viral-induced neuroinflammation.

**Methods:**

Acute encephalomyelitis and immune-mediated demyelination were induced by intracranial injection with the neurotropic JHM strain of mouse hepatitis virus (JHMV) into C57BL/6 *miR-155*^*+/+*^ wildtype (WT) mice or *miR-155*^*−/−*^ mice. Morbidity and mortality, viral load and immune cell accumulation in the CNS, and spinal cord demyelination were assessed at defined points post-infection. T cells harvested from infected mice were used to examine cytolytic activity, cytokine activity, and expression of certain chemokine receptors. To determine the impact of miR-155 on trafficking, T cells from infected WT or *miR-155*^*−/−*^ mice were adoptively transferred into *RAG1*^*−/−*^ mice, and T cell accumulation into the CNS was assessed using flow cytometry. Statistical significance was determined using the Mantel-Cox log-rank test or Student’s *T* tests.

**Results:**

Compared to WT mice, JHMV-infected *miR-155*^*−/−*^ mice developed exacerbated disease concomitant with increased morbidity/mortality and an inability to control viral replication within the CNS. In corroboration with increased susceptibility to disease, *miR-155*^*−/−*^ mice had diminished CD8^+^ T cell responses in terms of numbers, cytolytic activity, IFN-γ secretion, and homing to the CNS that corresponded with reduced expression of the chemokine receptor CXCR3. Both IFN-γ secretion and trafficking were impaired in *miR-155*^*−/−*^, virus-specific CD4^+^ T cells; however, expression of the chemokine homing receptors analyzed on CD4^+^ cells was not affected. Except for very early during infection, there were not significant differences in macrophage infiltration into the CNS between WT and *miR-155*^*−/−*^ JHMV-infected mice, and the severity of demyelination was similar at 14 days p.i. between WT and *miR-155*^*−/−*^ JHMV-infected mice.

**Conclusions:**

These findings support a novel role for miR-155 in host defense in a model of viral-induced encephalomyelitis. Specifically, miR-155 enhances antiviral T cell responses including cytokine secretion, cytolytic activity, and homing to the CNS in response to viral infection. Further, miR-155 can play either a host-protective or host-damaging role during neuroinflammation depending on the disease trigger.

## Background

MicroRNAs (miRNAs) are a new class of evolutionarily conserved gene-regulatory molecules that function to repress key target genes, primarily at the post-transcriptional level through specific mRNA 3′ untranslated region (3′ UTR) interactions [1]. Because miRNAs commonly target critical signaling proteins and transcription factors with potent regulatory impacts on the immune system [2, 3], it is accepted that miRNAs have an important effect on immune system activation and cellular differentiation. Recent work by our group and others has determined that miR-155 is an important regulator of immune cell development and function. Following its original identification as an oncogene in chicken lymphomas [4], miR-155 was discovered to be overexpressed in mammalian hematopoietic cancers and shortly thereafter established as an immunomodulatory noncoding RNA in macrophages and B lymphocytes [5–9]. It is now clear that miR-155 is expressed by and functions within a variety of activated immune cell types that include various T cell populations, NK cells, and dendritic cells [6, 7, 10–12]. In addition, miR-155 represses a variety of immunoregulatory proteins that include signaling molecules such as Ship1 [13] and Socs1 [14], as well as transcriptional regulators such as Jarid2 [15], Ets1 [16, 17], PU.1 [18], and Fosl2 [19].

Consistent with its known roles in regulating immune factors, multiple studies have demonstrated that miR-155 is important in shaping the immune responses that govern viral pathogenesis [20]. Genetic silencing of miR-155 results in increased sensitivity to experimental infection with lymphocytic choriomeningitis virus (LCMV) [21, 22], influenza virus [23], and herpes simplex virus (HSV) [24, 25]. While miR-155 had previously been shown to help tailor CD4^+^ T cell responses in models of autoimmunity, viral studies have since illustrated the importance of miR-155 in strengthening CD8^+^ T cell responses. Recent reports showed that miR-155 is required for optimal CD8^+^ T cell function following experimental infection with LCMV in terms of CTL activity, cytokine secretion, and proliferation [21, 22]. With regard to viral-induced encephalitis, miR-155 is important in controlling neuroinflammation, presumably by regulating T cell responses [24, 26]. These reports have emphasized the importance of miR-155 in augmenting host defense following viral infection; however, there have been few rigorous studies examining how miR-155 influences immune cell responses in a model of viral-induced encephalomyelitis.

Inoculation of the neurotropic JHM strain of mouse hepatitis virus (JHMV) into the CNS of susceptible strains of mice provides an excellent model for examining host response mechanisms responsible for controlling viral replication and modulating neuroinflammation within distinct cell lineages present in the brain [27, 28]. During acute disease, control of viral replication is mediated by infiltrating CD4^+^ and CD8^+^ T cells [29–31]; however, clearance of virus is not complete, and animals that survive the acute disease develop an immune-mediated demyelinating disease with both T cells and macrophages amplifying disease severity by contributing to myelin damage [32–38]. Our findings demonstrated that miR-155 was necessary for optimal T cell accumulation, cytolytic activity, cytokine secretion, and trafficking to the CNS after JHMV infection. Macrophage migration and accumulation within the CNS was not impaired in the absence of miR-155 during the time period studied, and there were no differences in the severity of demyelination at 14 days pi, when peak disease severity generally occurs. These results demonstrate that miR-155 has an important role in regulating antiviral T cell responses following viral-induced neuroinflammation.

## Methods

### Virus and mice

For intracranial (i.c.) injections, age-matched (5–7 weeks) C57BL/6 *miR-155*^*+/+*^ mice (wildtype (WT)) or *miR-155*^*−/−*^ mice were anesthetized with an intraperitoneal (i.p.) injection of 200 μl of a mixture of ketamine (Hospira, Lake Forest, IL, USA) and xylazine (Phoenix Pharmaceutical, Saint Joseph, MO, USA) in Hank’s balanced salt solution (HBSS). Mice were injected intracranially (i.c.) with 200 plaque-forming units (PFU) of JHMV (strain V34) suspended in 30 μl HBSS [39]. Clinical severity was assessed using a previously described four-point scoring scale [40]. For analysis of viral titers, mice were sacrificed at indicated time points. One half of each brain was homogenized and used in a plaque assay performed using the DBT mouse astrocytoma cell line [41]. The DM-JHMV (2.5 × 10^5^ PFU) strain [31, 42] was used to immunize experimental mice via i.p. injection to generate virus-specific T cells. This is an established and reliable method to accurately measure T cell responses following JHMV infection [42, 43]. *RAG1*^*−/−*^ mice were purchased from Jackson Laboratories. All animal studies were reviewed and approved by the University of Utah Animal Care and Use Committee.

### Cell isolation and flow cytometry

Immunophenotyping of immune cells present within brains and spinal cords of JHMV-infected mice at defined times post-infection (p.i.) was accomplished by homogenizing isolated tissue and generating single-cell suspensions for analysis by flow cytometry using previously described procedures [44–46]. In brief, isolated cells were stained with the following antibodies: APC-conjugated rat anti-mouse CD4 and a PE-conjugated tetramer specific for the CD4 immunodominant epitope present within the JHMV matrix (M) glycoprotein spanning amino acids 133-147 (M133-147 tetramer) to determine total and virus-specific CD4^+^ cells, respectively [47]; APC-conjugated rat anti-mouse CD8a and a PE-conjugated tetramer specific for the CD8 immunodominant epitope present in the spike (S) glycoprotein spanning amino acids 510-518 (S510-518) to identify total and virus-specific CD8^+^ cells, respectively; and APC-conjugated rat anti-mouse CD45 and FITC-conjugated anti-F4/80 to identify macrophages. Samples were analyzed using a BD LSR Fortessa X-20 flow cytometer and FloJo software.

### CD8^+^ T cell cytotoxicity assay

WT and *miR-155*^*−/−*^ mice were infected i.p. with the DM strain of JHMV (DM-JHMV, 2.5 × 10^5^ PFU), and a cytolytic T cell (CTL) assay was performed as previously described [31]. In brief, RMA-S target cells were seeded at a density of 10,000 cells/well in a flat-bottom 96-well tissue culture plate (Corning Life Sciences) and pulsed overnight with 50 μM OVA peptide or the immunodominant CD8 peptide specific for MHV spike (S) glycoprotein spanning amino acids 510-518 (S510-518, Bio-Synthesis). CD8^+^ T cells were isolated from splenocytes of infected mice at day 8 p.i. using MACS® Separation Columns and CD8^+^ T cell Isolation kit (Miltenyi). Equivalent numbers of virus-specific CD8^+^ T cells from WT and *miR-155*^*−/−*^ mice were then incubated with RMA-S cells at different effector-to-target (E:T) ratios. Co-cultures were incubated for 4 h at 37 °C in 5 % CO_2_ at a final volume of 200 μL/well. The levels of lactate dehydrogenase released from lysed cells were determined using a LDH Cytotoxicity Assay Kit (Pierce). The percentage of CTL-mediated lysis was determined as specified by the manufacturer’s specifications as previously described [31].

### IFN-γ production

CD4^+^ and CD8^+^ T cells were isolated from the spleens of WT and *miR-155*^***−/−***^ mice infected i.p. with DM-JHMV (2.5 **×** 10^5^ PFU) and used to assess cytokine secretion in response to defined viral epitopes [39]. CD4^+^ and CD8^+^ T cells were isolated as described above using an isolation kit according to the manufacturer’s instructions (Miltenyi Biotec, Auburn, CA, USA). Enriched T cell subsets (1 **×** 10^6^ cells) were incubated with antigen-presenting cells in flat-bottom 96-well plates and incubated for 24 h at 37 °C in 5 % CO_2_ in the presence of 5 μM of either OVA, M133, or S510 peptides. Supernatants were collected and IFN-γ levels were measured using ELISA (R & D Systems, Minneapolis, MN, USA).

### Histology

Spinal cords were isolated at defined time points and fixed overnight with 4 % paraformaldehyde at 4 °C. Sections were subsequently cryoprotected in 30 % sucrose for 5–7 days, separated into 12 coronal sections, and embedded in optimum cutting temperature (OCT) formulation (VWR, Radnor, PA, USA) [48]. Coronal sections (8 μm thick) were cut, and sections were stained with luxol fast blue (LFB) in combination with hematoxylin and eosin (H & E). Areas of total white matter and demyelinated white matter were determined with Image J Software. The percent demyelination was calculated by dividing the area of demyelinated white matter by the total white matter area using established methods previously described [47, 49].

### Adoptive transfer

Adoptive transfer experiments were performed using previously described protocols [42]. In brief, WT and *miR-155*^*−/−*^ mice were injected i.p. with JHMV-DM (2.5 × 10^5^ PFU) and spleens removed at day 7 p.i.. CD4^+^ and CD8^+^ T cells were enriched via magnetic separation (Miltenyi) and equal numbers of virus-specific T cells (determined by tetramer staining) were adoptively transferred via intravenous (i.v.) injection into the retro-orbital sinus of *RAG1*^*−/−*^ mice 3 days following i.c. infection with 200 PFU of JHMV. Mice were sacrificed 9 days post-transfer (12 days p.i.), and brains and spinal cords were removed. One half of the brains were used for flow cytometry analysis, and the remaining halves were used to determine viral titers. Control animals included JHMV-infected *RAG-1*^*−/−*^ mice.

## Results

### Increased disease severity in JHMV-infected *miR-155*^*−/−*^ mice

**Fig. 1:**
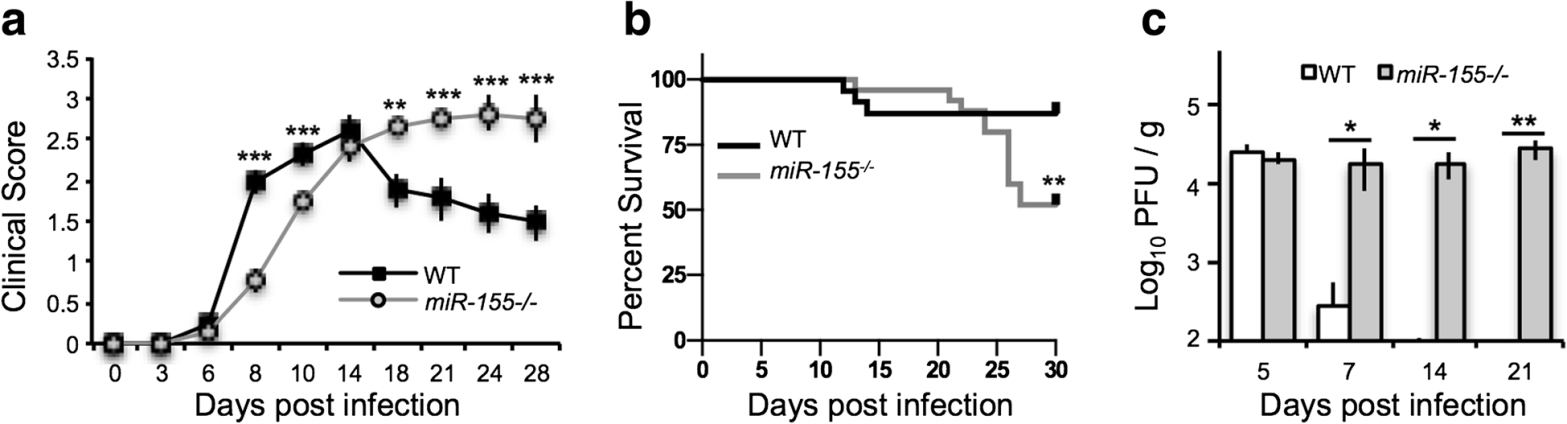
Increased morbidity/mortality in JHMV-infected *miR-155* ^*−/−*^ mice was associated with elevated viral titers within the brain. WT (*n* = 12) and *miR-155* ^*−/−*^ mice (*n* = 12) were infected via i.c. injection with 200 PFU of JHMV. Clinical scores (**a**) and survival (**b**) were assessed throughout infection. The increase in both clinical disease and mortality correlated with an impaired ability to control viral replication within the brains at the indicated times p.i. **c** Statistical significance was determined using Mantel-Cox log-rank test or one-tailed, unpaired, Student’s *T* tests. Data are representative of at least two independent experiments; ^*^ *p* < 0.05; ^**^ *p* < 0.01; ^***^ *p* < 0.001

Age-matched WT or *miR-155*^*−/−*^ mice were i.c. inoculated with JHMV (200 PFU), and the severity of clinical disease and survival were monitored. JHMV-infected *miR-155*^*−/−*^ mice demonstrated delayed onset of disease compared to WT mice, yet clinical disease was sustained in *miR-155*^*−/−*^ animals compared to WT mice (Fig. 1a). By day 30 p.i., 85 % of WT and 54 % of *miR-155*^*−/−*^ mice had survived (Fig. 1b). Assessment of viral titers within the brains of infected mice revealed similar titers 5 days p.i.; however, by day 7 p.i., WT mice had dramatically reduced viral titers, and by day 14 p.i., titers were below the level of detection (~100 PFU/g) (Fig. 1c). In contrast, JHMV-infected *miR-155*^*−/−*^ mice were unable to control viral replication and demonstrated high viral titers out to 21 p.i. (Fig. 1c). Collectively, these data indicate that miR-155 expression enhances immune-mediated control of viral replication within the CNS.

### Impaired T cell response in JHMV-infected *miR-155*^*−/−*^ mice

**Fig. 2:**
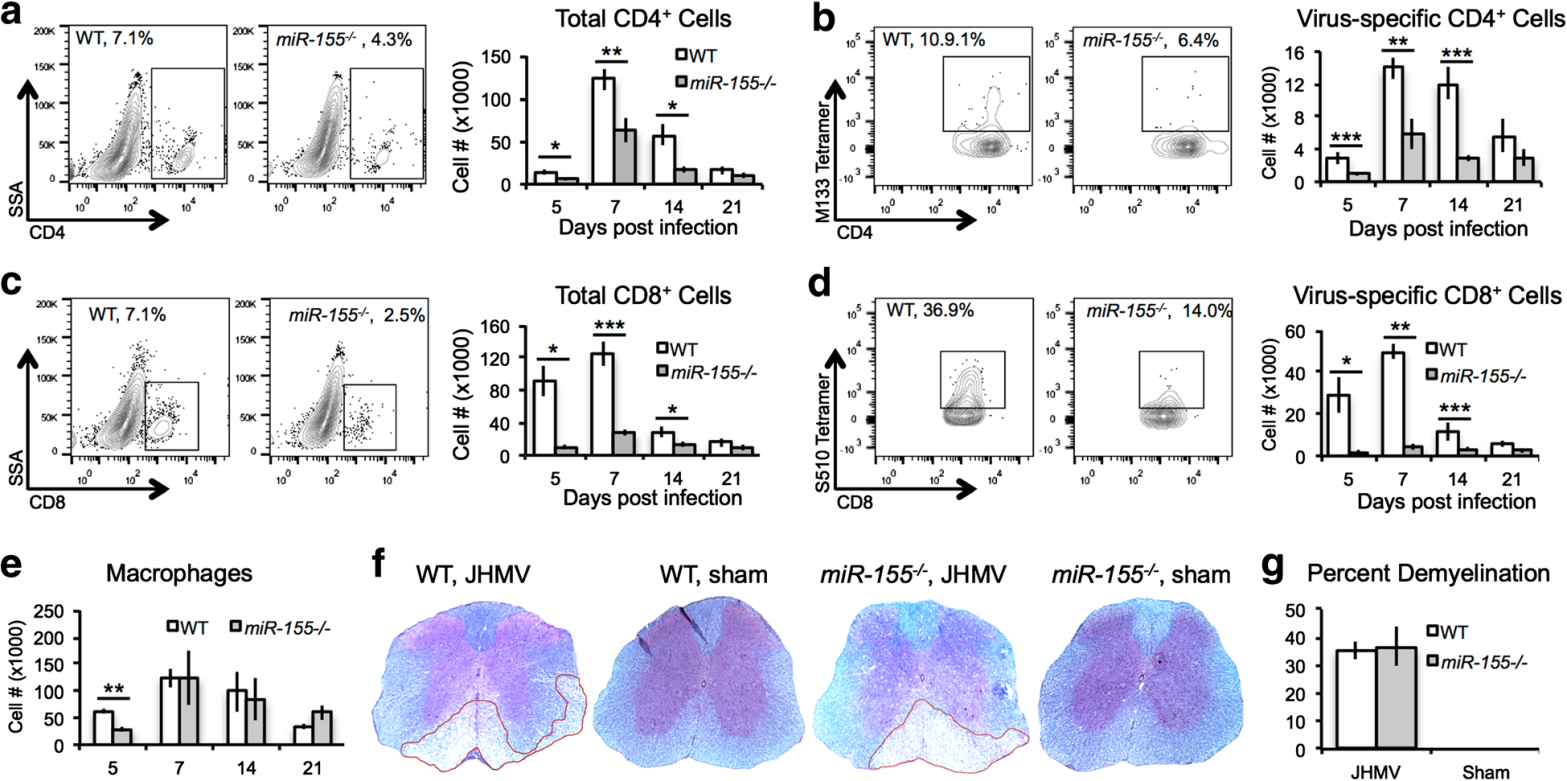
JHMV-infected *miR-155* ^*−/−*^ mice demonstrated reduced CNS T cell infiltration. WT and *miR-155* ^*−/−*^ mice were infected i.c. with JHMV (200 PFU). Mice from each group were sacrificed 5, 7, 14, and 21 days p.i., and brains were collected. Flow analysis indicated reduced infiltration of total CD4^+^ T cells (**a**) and CD8+ T cells (**c**), as well as reduced virus-specific CD4^+^ T cells (**b**) and CD8+ T cells (**d**), as determined by tetramer staining [95, 96]. In contrast, while macrophage (CD45 + F4/80^hi^) infiltration into the CNS was lower in *miR-155* ^*−/−*^ mice at 5 days p.i. (**e**), the levels were similar at later time points. Representative spinal cords from JHMV-infected and sham-infected mice stained with LFB at day 14 p.i. showed similar levels of demyelination between infected WT mice (35.1 + 4.9 %, *n* = 4) and *miR-155* ^*−/−*^ mice (36.7 + 4.3, *n* = 4) whereas no demyelination is observed in sham-infected animals (**f**, **g**). Data presented are derived from two independent experiments with a minimum of four mice/experimental group. Data are presented as average ± SEM. Statistical significance was measured using unpaired, one-tailed Student’s *T* tests; ^*^ *p* < 0.05; ^**^ *p* < 0.01; ^***^ *p* < 0.001

T cell responses are critical for controlling JHMV replication within the CNS [27, 29, 50–57]. Therefore, we next wished to determine whether increased morbidity/mortality and inability to control viral replication correlated with impaired T cell accumulation within the brains of JHMV-infected *miR-155*^*−/−*^ mice. Brains were harvested from JHMV-infected WT or *miR-155*^*−/−*^ mice at 5, 7, 14, and 21 days p.i., and immune cells were isolated and immunophenotyped using flow cytometry [47, 58, 59]. Both total CD4^+^ and virus-specific CD4^+^ cells were decreased in brains from *miR-155*^*−/−*^ mice compared to WT mice at 5, 7, and 14 days p.i. (Fig. 2a, b); however, by 21 days p.i., no differences were detected. In addition, levels of total and virus-specific CD8^+^ cells were dramatically decreased in brains from *miR-155*^*−/−*^ mice compared to WT mice at 5, 7, and 14 days p.i. yet not at day 21 p.i. (Fig. 2c, d). There were decreased levels of macrophages at 5 days p.i. in brains of *miR-155*^*−/−*^ mice compared to WT mice; however, there were no significant differences in CNS macrophage accumulation at later times (Fig. 2e). The degree of demyelination at day 14 p.i. was similar between JHMV-infected WT (35.1 ± 4.9 %, *n* = 4) and *miR-155*^*−/−*^ mice (36.7 % ± 4.3 %, *n* = 4) (Fig. 2f, g). These results demonstrate that miR-155 is necessary for optimal T cell accumulation in the CNS in the context of JHMV infection, and is consistent with previous studies.

**Fig. 3:**
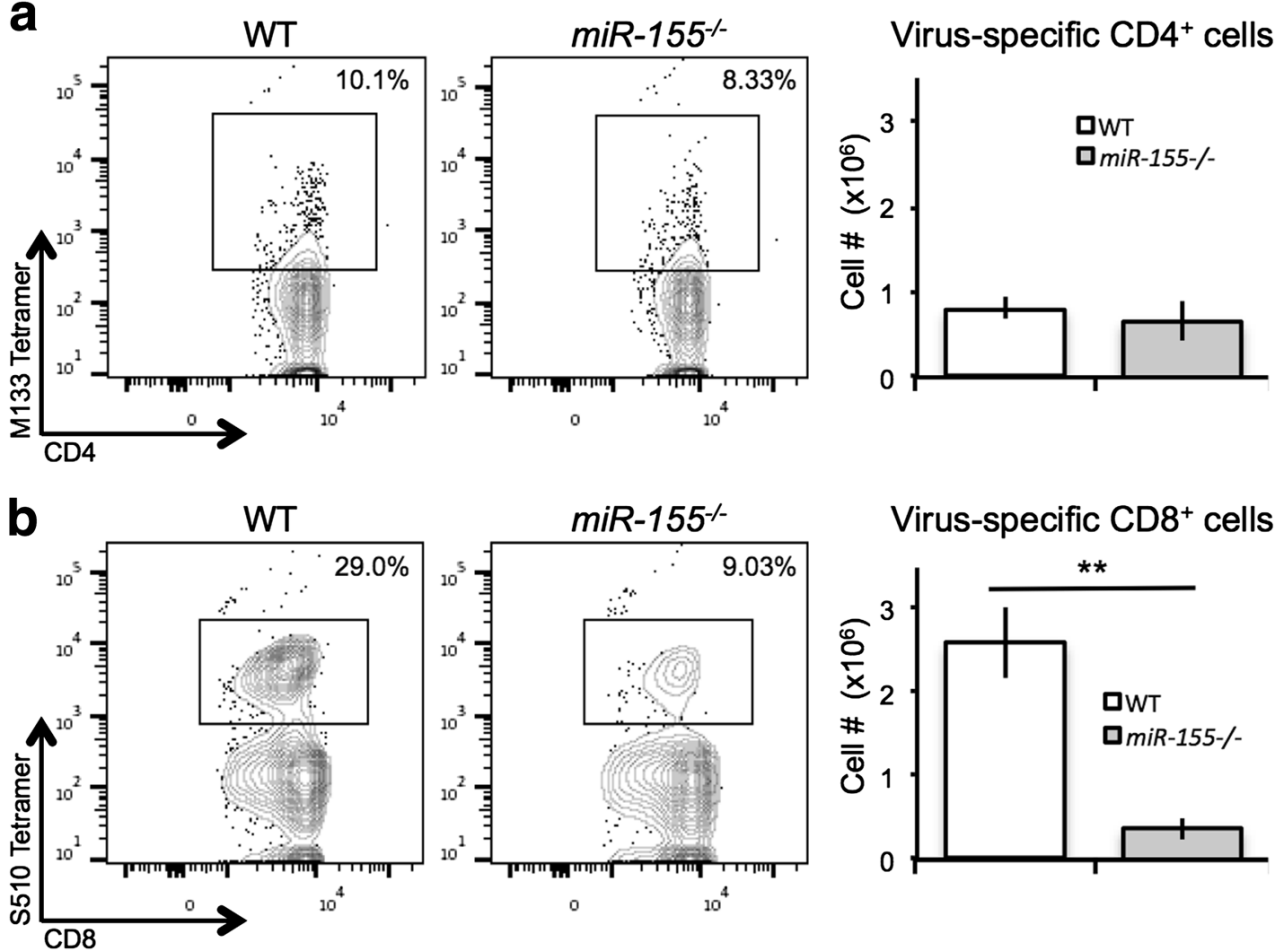
miR-155enhanced expansion of virus-specific CD8^+^ T cells. WT and *miR-155* ^*−/−*^ mice were i.p. infected with DM-JHMV. Spleens were removed at day 8 p.i., and virus-specific T cells were identified by tetramer staining. Representative dot blots indicated that while similar frequencies and numbers of virus-specific CD4^+^ T cells (**a**) were present in WT and *miR-155* ^*−/−*^ mice, there was a significant (**p < 0.001) decrease in both the frequency and numbers of virus-specific CD8^+^ T cells (**b**) in *miR-155* ^*−/−*^ mice compared to WT mice. Histograms are presented as average ± SEM; statistical significance was measured using unpaired, one-tailed Student’s *T* tests. Data presented are derived from two independent experiments with a minimum of four mice/experimental group. ^*^ *p* < 0.05; ^**^ *p* < 0.01; ^***^ *p* < 0.001

These findings suggest that the absence of miR-155 during acute viral-induced encephalomyelitis affects either the ability of virus-specific T cells to expand and/or traffic to the CNS [45, 46]. To test whether miR-155 affects expansion in the context of JHMV infection, we immunized WT and *miR-155*^*−/−*^ mice by intraperitoneal (i.p.) injection with 2.5 × 10^5^ PFU of DM-JHMV [31, 42] and isolated splenocytes at day 8 p.i. to determine the frequency and number of virus-specific T cells by tetramer staining. Similar numbers of M133-147 virus-specific CD4^+^ T cells were present in *miR-155*^*−/−*^ mice compared to WT (Fig. 3a). In contrast, there was a significant (*p* < 0.001) decrease in S510-518-specific CD8^+^ T cells in splenocytes from *miR-155*^*−/−*^ mice compared to those from WT mice (Fig. 3b), indicating that miR-155 is necessary for optimal CD8^+^ T cell expansion.

### Antiviral T cell activity is muted in *miR-155*^*−/−*^ mice

**Fig. 4:**
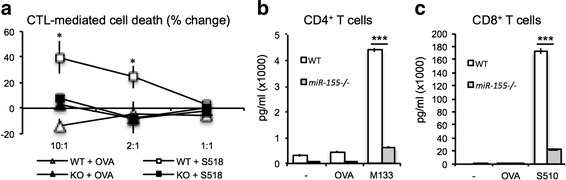
T cells from *miR-155* ^*−/−*^ mice exhibited impaired antiviral effector function. WT and *miR-155* ^*−/−*^ mice were immunized with DM-MHV via i.p. injection. **a** Animals were sacrificed 8 days p.i., and CD4^+^ and CD8^+^ T cells were isolated and pooled. The frequencies of total and virus-specific CD8^+^ T cells were determined by tetramer staining [96]. Equivalent numbers of virus-specific CTLs were added to target cells pulsed with either the immunodominant CD8^+^ T cell epitope within the spike (S) glycoprotein spanning residues 510-518 (S510-518, 50 μM) or control ovalbumin peptide (50 μM) at the indicated ratios, and lytic activity was determined as previously described [31, 96]. In addition, antigen recall responses to the immunodominant CD4 T cell epitope (M133-147) (**b**) or CD8 T cell epitope S510-518 (**c**) was performed, and IFN-γ levels were determined by ELISA as previously described [96]. Data are representative of at least two independent experiments, with at least five mice from each group. ^*^ *p* < 0.05; ^***^ *p* < 0.001

We next examined whether T cell antiviral effector responses were altered in the absence of miR-155 expression. Both cytolytic activity by CD8^+^ T cells [27, 50, 57, 60], as well as secretion of IFN-γ by virus-specific CD4^+^ and CD8^+^ T cells are important for controlling JHMV replication within the CNS [31, 53, 54, 61, 62]. WT and *miR-155*^*−/−*^ mice were infected i.p. with DM-JHMV. Splenocytes were removed 8 days p.i., and the antiviral activity of virus-specific T cells was determined. As shown in Fig. 4a, virus-specific, *miR-155*^*−/−*^ CD8^+^ T cells showed reduced (*p* < 0.05) cytolytic activity compared to WT CD8^+^ T cells. In addition, secretion of IFN-γ by CD4^+^ and CD8^+^ T cells from immunized *miR-155*^*−/−*^ mice was reduced (*p* < 0.001) compared to WT mice (Fig. 4b, c). These findings argue that in the absence of miR-155, virus-specific T cell functions are blunted, consistent with previous reports [21, 22, 24, 25].

### miR-155 ablation impairs T cell migration to the CNS of JHMV-infected *RAG-1*^*−/−*^ mice

**Fig. 5:**
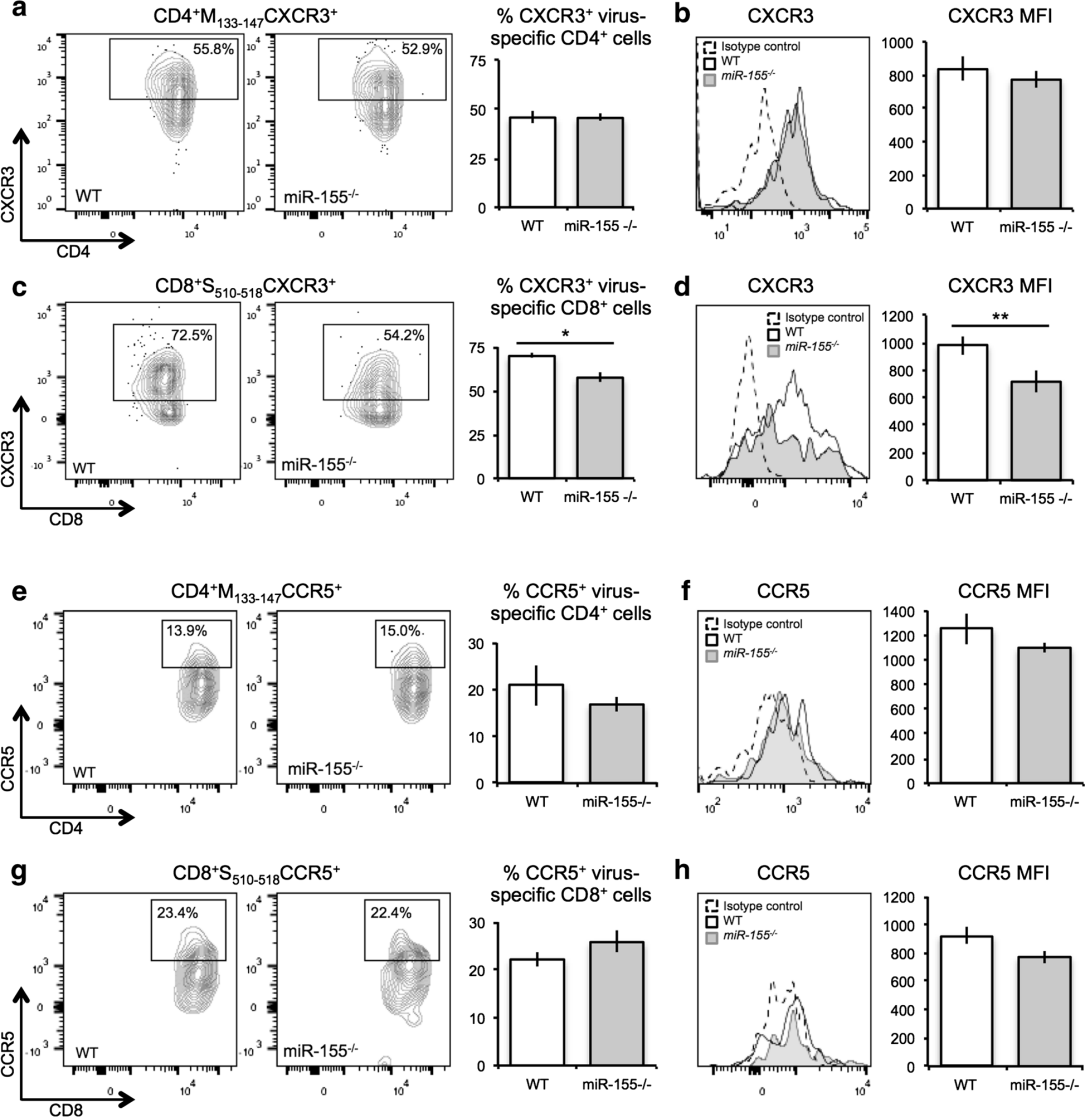
Chemokine receptor expression on virus-specific WT and *miR-155* ^*−/−*^ mice. WT and *miR-155* ^*−/−*^ mice were infected i.p. with DM-MHV and spleens were isolated at day 8 p.i.. Flow cytometric analysis revealed similar levels of CXCR3 on virus-specific CD4^+^ T cells isolated from WT and *miR-155* ^*−/−*^ mice (**a**, **b**). However, expression of CXCR3 was decreased (*p* < 0.05) on virus-specific CD8^+^ T cells isolated from *miR-155* ^*−/−*^ mice compared to WT mice (**c**, **d**). Flow cytometric analysis revealed similar levels of CCR5 on both virus-specific CD4^+^ T cells (**e**, **f**) and virus-specific CD8^+^ T cells (**g**, **h**) isolated from WT and *miR-155* ^*−/−*^ mice. Plots represent average ± SEM; statistical significance was measured using unpaired, one-tailed Student’s *T* tests. Data are representative of two independent experiments, with a minimum of four mice per group per experiment. ^*^ *p* < 0.05; ^**^ *p* < 0.01

Our findings indicate that in the absence of miR-155, antiviral T cell responses are dampened following JHMV infection of the CNS. In addition, the inability to control viral replication within the CNS was associated with fewer numbers of T cells within the CNS of JHMV-infected *miR-155*^*−/−*^ mice compared to infected WT mice, raising the possibility of a deficiency in T cell homing in the absence of miR-155. We have previously shown that T cell expression of the chemokine receptor CXCR3, the signaling receptor for the chemokine CXCL10, is important in enhancing the ability of these cells to migrate and accumulate within the CNS of JHMV-infected mice [44, 45, 63–65]. We therefore tested whether expression of CXCR3 was decreased on T cells from JHMV-infected *miR-155*^*−/−*^ mice. There were no differences in expression of CXCR3 on M133-147-specific CD4^+^ T cells (Fig. 5a, b). In contrast, there was an overall reduction (*p* < 0.05) in the frequency of CXCR3-positive S510-518-positive CD8^+^ T cells (Fig. 5c), as well as a reduction (*p* < 0.01) of CXCR3 on a per-cell level (Fig. 5d). These findings indicate that miR-155 regulates expression of CXCR3 on CD8^+^ T cells, and this corresponds with impaired trafficking of these cells to the CNS following JHMV infection. The paucity in CD4^+^ T cell trafficking to the CNS of JHMV-infected *miR-155*^*−/−*^ mice suggests the possibility that other T cell homing receptors such as CCR5 may be affected by miR-155 deficiency and account for impaired CNS migration [66]; however, analysis of CCR5 on virus-specific CD4^+^ (Fig. 5e, f) and CD8^+^ T cells (Fig. 5g, h) indicated no differences in surface expression of this homing receptor between WT and *miR-155*^*−/−*^ T cells.

**Fig. 6:**
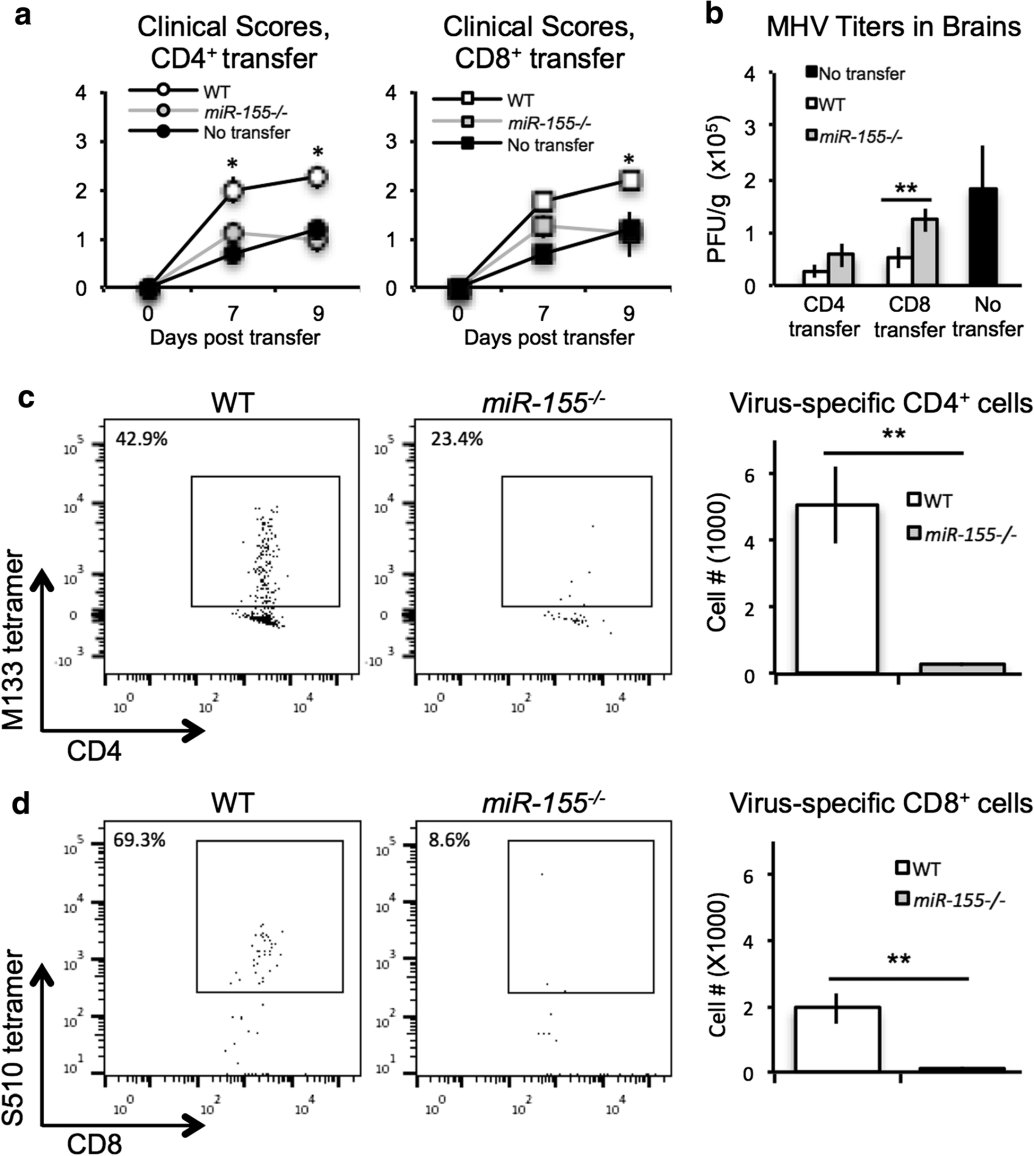
Silencing of miR-155 dampened the accumulation of adoptively transferred virus-specific T cells within the CNS of JHMV-infected *RAG-1* ^*−/−*^ mice. JHMV-infected *RAG1* ^*−/−*^ mice received equal numbers of either WT or *miR-155* ^*−/−*^ virus-specific CD4^+^ or CD8^+^ T cells via i.v. injection on the day following i.c. instillation of virus. **a** Clinical disease in mice that received either *miR-155* ^*−/−*^ CD4^+^ (*n* = 3) or CD8^+^ T cells (*n* = 3) was reduced (*p* < 0.05) when compared to recipients of WT CD4^+^ (*n* = 3) or CD8^+^ (*n* = 3) cells. Increased disease severity was associated with an inability to control viral replication within the CNS (**b**) and a dramatic reduction (*p* < 0.01) in migration of CD4^+^ (**c**) and CD8^+^ (**d**) T cells into the brains compared to WT cells at day 9 post-transfer. **a**, **b** Data are presented as average ± SEM. **c**, **d** Representative dot blots are shown and histograms are presented as average ± SEM. Statistical significance was measured using unpaired, one-tailed Student’s *T* tests. ^*^ *p* < 0.05; ^**^ *p* < 0.01

As an additional test to determine if the absence of miR-155 affected T cell migration into the CNS, we performed adoptive transfer experiments. WT and *miR-155*^*−/−*^ mice were injected i.p. with DM-MHV. Eight days p.i., spleens were isolated and equal numbers of virus-specific CD4^+^ or CD8^+^ T cells from WT or *miR-155*^*−/−*^ mice were injected i.v. into *RAG-1*^*−/−*^ mice (deficient in functional T and B lymphocytes) that had been infected i.c. with JHMV 3 days prior. As shown in Fig. 6a, JHMV-infected *RAG-1*^*−/−*^ recipients of either virus-specific CD4^+^ or CD8^+^ T cells from WT mice showed increased (*p* < 0.05) clinical disease severity compared to recipients of *miR-155*^*−/−*^ T cells. Animals were sacrificed at day 9 post-transfer (day 12 p.i.), and viral titers and T cell infiltration into the CNS were assessed. Our findings indicate that viral titers within the brain were higher in JHMV-infected *RAG1*^*−/−*^ mice that received either virus-specific *miR-155*^*−/−*^ CD4^+^ or CD8^+^ T cells compared to recipients of WT virus-specific T cell subsets (Fig. 6b). Importantly, CNS accumulation of both virus-specific CD4^+^ (Fig. 6c) and CD8^+^ (Fig. 6d) T cells was significantly (*p* < 0.01) reduced in mice that received *miR-155*^*−/−*^ T cells compared to recipients of WT T cells. These results provide further evidence that miR-155 is important for T cell trafficking.

## Discussion

In this report, we have examined the mechanisms by which miR-155 affects both host defense and disease progression following JHMV infection of the CNS. Our findings revealed that miR-155 expression is associated with susceptibility to JHMV-induced neurologic disease. Expression of miR-155 is necessary for effective antiviral T cell responses as ablation of miR-155 resulted in increased morbidity/mortality that was associated with elevated viral titers within the CNS. Increased disease severity most likely reflects dampened CD8^+^ T cell responses, as reflected by reduced CNS accumulation of virus-specific CD8^+^ T cells. Furthermore, cytolytic activity by CD8^+^ T cells, as well as secretion of IFN-γ, was reduced in *miR-155*^*−/−*^ CD8^+^ T cells, highlighting a role for this molecule in configuring effective responses by virus-specific CD8^+^ T cells. While expansion of virus-specific CD4^+^ T cells was not affected in the absence of miR-155, IFN-γ secretion by CD4^+^ T cells was diminished. Importantly, the ability of T cells to migrate to the CNS was dramatically reduced in the absence of miR-155 expression, and this was associated with increased susceptibility to JHMV-induced neurologic disease. Whether increased susceptibility to JHMV-induced neurologic disease reflects a T cell intrinsic problem or whether our findings reflect an extrinsic effect via other immune cells, e.g., dendritic cells, is not known at this time. While this is an important question, we believe that muted antiviral T cell responses in *miR-155*^*−/−*^ mice following JHMV infection reflects an intrinsic problem in that (i) adoptive transfer of *miR-155*^*−/−*^ virus-specific T cells into JHMV-infected mice was unable to effectively reduce CNS viral titers and (ii) recent studies employing experimental infection of *miR-155*^*−/−*^ mice with lymphocytic choriomeningitis virus (LCMV) indicate that impaired T cell responses are due to specific deficiencies in T cells [21].

Within the context of neuroinflammatory diseases, miR-155 was initially shown to be critical in the induction of myelin-reactive Th17 cells in EAE, the prototypic model of the human demyelinating disease multiple sclerosis (MS) [67, 68]. In addition, miR-155 expression by endothelial cells of the blood-brain barrier (BBB) has been shown to regulate BBB function and affect neuroinflammation during EAE [69]. More recently, a role for miR-155 has been implicated in contributing to neuroinflammation in models of Parkinson’s disease [70], Alzheimer’s disease [71], alcohol-induced neuroinflammation [72], and amyotrophic lateral sclerosis (ALS) [73]. Although the mechanisms by which miR-155 affects neuroinflammation have not been firmly established, an emerging concept is that expression of miR-155 by microglia is important in regulating expression of proinflammatory genes that subsequently influence neuroinflammation [74–77]. We are currently further investigating the mechanisms by which miR-155 affects host defense and disease progression in models of viral encephalitis. Results from the current study are congruent with recent reports by Rouse and colleagues [24] demonstrating that miR-155 affects susceptibility to HSV-1-induced encephalitis as a result of impaired antiviral T cell responses as well as homing to the CNS. Similarly, the severity of neuroinflammation is reduced following experimental infection with Japanese encephalitis virus (JEV) in the absence of miR-155, and this is associated with dampened expression of proinflammatory cytokines [26]. These findings emphasize an important role for miR-155 in augmenting host defense in response to CNS infection by neurotropic viruses through different mechanisms, including regulating gene expression by resident glia and tailoring T cell responses. With regard to the former, negative regulation of Ship1 by miR-155 was found to affect expression of proinflammatory cytokines and modulate neuroinflammation during JEV infection [26]. A number of different mechanisms by which miR-155 controls T cell responses following viral infection have been proposed. In the absence of miR-155, virus-specific CD8^+^ T cells have enhanced type-I interferon signaling, leading to increased susceptibility to interferon’s anti-proliferative effect [23]. Impaired antiviral CD8^+^ T cell responses have also been associated with reduced activation of the prosurvival Akt pathway, arguing that miR-155 promotes T cell survival/function in response to viral infection [21]. Targeting of Socs1 by miR-155 has also been shown to disrupt T cell function in response to viral infection, and these studies emphasized the importance of both cell type and context in determining how miR-155 affects lymphocyte function [22]. Whether these miR-155-related pathways and/or targets are affected in response to JHMV infection of the CNS remains to be determined and is the focus of ongoing studies by our group.

Previous work from our lab and others has implicated chemokines as important in regulating lymphocyte migration to the CNS in response to viral infection [78]. Specifically, we have shown that expression of both CXCR3 and CCR5 promote migration of virus-specific T cells into the CNS of JHMV-infected mice [42, 43, 45, 79]. Our findings that impaired migration of miR-155-deficient, virus-specific CD8+ T cells to the CNS of JHMV-infected mice correlated with reduced expression of CXCR3, but not CCR5, are interesting and argue that expression of chemokine homing receptors may be modulated by miR-155. In our hands, this effect was restricted to CD8^+^ T cells, as neither CXCR3 nor CCR5 expression was affected in miR-155-deficient CD4^+^ T cells. Nonetheless, homing to the CNS by CD4^+^ cells was reduced, arguing that the absence of miR-155 may affect the ability of these cells to efficiently migrate to sites of infection. This theory was further supported by adoptive transfer experiments demonstrating that in *RAG-1*^*−/−*^ mice that received miR-155-deficient CD4^+^ or CD8^+^ cells, there was a dramatic deficiency in CNS accumulation of CD4^+^ or CD8^+^ T cells, respectively. In addition, there was an impaired ability to control viral replication compared to recipients of WT cells. Recent work has demonstrated that miRNAs, including miR-155, may influence chemokine receptor expression on circulating lymphocytes [80–82], suggesting that sufficient expression of these homing receptors is intrinsically influenced by miRNAs.

Mice persistently infected with JHMV develop an immune-mediated demyelinating disease in which chronic infiltration of virus-specific T cells and macrophages amplifies the severity of demyelination. The profile of clinical symptoms and accompanying histopathology associated with JHMV persistence has been employed as a pre-clinical animal model of the human demyelinating disease multiple sclerosis (MS) [28, 83, 84]. Previous studies have demonstrated that genetic silencing of miR-155 ameliorates the severity of EAE and this was associated with a reduction in the severity of neuroinflammation and demyelination, highlighting that miR-155 has a functional role in pre-clinical MS models [67, 68]. Clinical studies in MS patients have suggested that microRNAs may be used as novel diagnostic and predictive biomarkers, as well as affect disease progression [85–87]. Evidence demonstrating a potentially important role for miR-155 in MS includes demonstration that miR-155 expression is increased in peripheral blood mononuclear cells [88] as well as in brain lesions [89] of MS patients. In addition, glatiramer acetate treatment resulted in normalization of deregulated miRNAs, including miR-155, in peripheral blood mononuclear cells in patients with relapsing-remitting MS, arguing that miR-155 has a role in the regulation of immune responses in MS patients [90]. Other potential roles for miR-155 in controlling disease progression include regulation of proinflammatory responses in blood-derived and CNS-resident myeloid cells [91]. Furthermore, microRNAs may represent novel regulators of oligodendrocyte differentiation via control of transcriptional networks that influence myelin gene expression and cell cycle transitions [92, 93]. Our findings indicate that in the JHMV model, miR-155 does not affect demyelination per se, as there were similar levels of myelin damage in JHMV-infected WT and *miR-155*^*−/−*^ mice at the peak of disease. Whether these results reflect the use of the JHM strain of MHV is not known at this time. The A59 strain of MHV has been shown to induce demyelination in the absence of the adaptive immune suggesting that macrophage/microglia may be sufficient to initiate white matter damage [94]. We are currently investigating whether miR-155 influences processes governing demyelination and/or remyelination at later stages of JHMV through control of oligodendrocyte progenitor maturation. In addition, we are examining whether the absence of miR-155 affects proinflammatory gene expression by resident glia, e.g., astrocytes and microglia.

## Conclusions

This study demonstrates that miR-155 contributes to antiviral T cell responses in a model of viral-induced encephalomyelitis. Our findings illustrate that the absence of miR-155 increases susceptibility to death in response to viral infection of the CNS and that this correlates with increased viral replication within the CNS, limited T cell trafficking to the CNS, muted secretion of IFN-γ, and reduced cytolytic activity. However, macrophage trafficking and the severity of demyelination were not significantly affected in virally infected *miR-155*^*−/−*^ mice, indicating increased disease severity reflected impaired T cell responses. Importantly, because miR-155 plays a host-protective role during JHMV-mediated neuroinflammation, yet plays a pathogenic role in autoimmune models of neuroinflammation and demyelination following immunization with encephalitogenic peptides, its therapeutic targeting in the clinic should be carefully considered.

## Abbreviations

CNS: Central nervous system
CTL: Cytotoxic T lymphocyte
ELISA: Enzyme-linked immunosorbent assay
FBS: Fetal bovine serum
H & E: Hematoxylin and eosin
i.c.: Intracranial
i.p.: Intraperitoneal
IFNγ: Interferon-gamma
LDH: Lactate dehydrogenase
LFB: Luxol fast blue
miR-155: microRNA 155
MRI: Magnetic resonance imaging
p.i.: Post-infection
PFU: Plaque-forming units
